# Barcoding utility in a mega-diverse, cross-continental genus: keeping pace with *Cyrtodactylus* geckos

**DOI:** 10.1038/s41598-017-05261-9

**Published:** 2017-07-17

**Authors:** Ian G. Brennan, Aaron M. Bauer, Ngo Van Tri, Yun-yu Wang, Wen-zhi Wang, Ya-Ping Zhang, Robert W. Murphy

**Affiliations:** 1 Division of Ecology & Evolution, Research School of Biology, The Australian National University, Canberra, ACT 2602 Australia; 2 National Key Laboratory, Institute of Tropical Biology, Vietnamese Academy of Sciences and Technology, 9/621 Hanoi Highway, Linh Trung District, Ho Chi Minh City, Vietnam; 3 State Key Laboratory of Genetic Resources and Evolution State, and Yunnan Laboratory of Molecular Biology of Domestic Animals, Kunming Institute of Zoology, Chinese Academy of Sciences, Kunming, 650223 China; 4 South China DNA Barcoding Center, Kunming Institute of Zoology, Chinese Academy of Sciences, Kunming, 650223 China; 5 Laboratory for Conservation and Utilization of Bio-resource and Key Laboratory for Microbial Resources of the Ministry of Education, Yunnan University, 2 Cuihu N. Rd., Kunming, 650091 China; 6 Centre for Biodiversity and Conservation Biology, Royal Ontario Museum, 100 Queen’s Park, Toronto, M5S 2C6 Canada

## Abstract

Over the past decade, DNA barcoding has become a staple of low-cost molecular systematic investigations. The availability of universal primers and subsidized sequencing projects (PolarBOL, SharkBOL, SpongeBOL) have driven this popularity, often without appropriate investigation into the utility of barcoding data for the taxonomic group of interest. Here, our primary aim is to determine the phylogenetic value of DNA barcoding (mitochondrial locus *COI*) within the gecko genus *Cyrtodactylus*. With >40 new species described since last systematic investigation, *Cyrtodactylus* represents one of the most diverse extant squamate genera, and their contemporary distribution spans the Indian subcontinent, eastward through Indochina, and into AustraloPapua. The complex biogeographic history of this group, and morphology-only designation of many species have complicated our phylogenetic understanding of *Cyrtodactylus*. To highlight the need for continued inclusive molecular assessment, we use Vietnamese *Cyrtodactylus* as a case study showing the geopolitically paraphyletic nature of their history. We compare *COI* to the legacy marker *ND2*, and discuss the value of *COI* as an interspecific marker, as well as its shortcomings at deeper evolutionary scales. We draw attention back to the Cold Code as a subsidized method for incorporating molecular methods into species descriptions in the effort to maintain accurate phylogenies.

## Introduction

## Barcoding the Tree of Life

Barcoding initiatives across the tree of life have helped document and describe thousands of species of bony fishes, birds, sharks, and sponges, among many other groups^1–5^. Cold Code^6^, the barcoding initiative for amphibians and non-avian reptiles, has similarly produced an immense quantity of sequence data for the mitochondrial locus encoding cytochrome c oxidase subunit I (*COI*). Cold Code and other barcoding initiatives provide a cost-free sequencing service for up to ten individuals of any species. In conjunction with databases such as the Barcode of Life Data Systems (BOLD), GenBank, and Dryad, researchers without access to sequencing facilities can produce and visualize novel sequences before adding preexisting data and running analyses. Implementation of Cold Code has contributed considerably to taxonomic resolution in Third World nations, and has been applied for conservation efforts in these regions that most need them^7^. Although Cold Code instigated barcoding on the grounds of species identification and discovery^8^, recent studies have increasingly used barcoding data for phylogenetic inference and to answer phylogeographic questions^9, 10^. This practice is often undertaken without sufficient assessment of the utility of barcoding for the taxonomic group of interest. Inference at deep timescales, may be severely compromised by the rapid mutational rate and limited size of the *COI* fragment used for barcoding. At shallower timescales, and in narrower phylogenetic contexts, DNA barcoding remains valuable^11^.

## Limitations to Barcoding

Despite ease of amplification, subsidized sequencing, and fast mutational rates making for high informativeness, mtDNA species-level inference via barcoding has its drawbacks. Mitochondrial phylogenetic reconstruction may be hampered by introgression and hybridization, male-biased gene flow, and selection on the linked mitochondrial genome, among other limitations^12^. Specifically, in several taxonomic groups—blowflies^13^; birds^14^; orthopterans^15^; dipterans^16^—mtDNA divergence and barcoding have been shown to be insufficient in delineating rapidly evolving species lineages, or those likely to introgress mitogenomes. However, these cases are interesting exceptions and when barcoding is used in concert with alternative methodologies such as ecology, morphology, and nuclear genomic data, barcoding is a powerful tool^17–19^. These integrative approaches facilitate pluralistic assessments of species delimitation and enhance accuracy. Requisite morphological diagnosis as part of species descriptions can quickly and easily pair with molecular data produced by DNA barcoding^20, 21^.

## Systematics of *Cyrtodactylus* Gray 1827

Since the last extensive molecular phylogenetic assessment of *Cyrtodactylus* ^22^, more than 40 new species have been described using morphological, molecular, or integrative methods^21, 23–25^. Indeed, as of 2016, several species^26–31^ and many lineages await description^23, 32, 33^. These add to the more than 200 formally described species^34^, and contribute to the growing number of publications (100+ per year) discussing *Cyrtodactylus* (Supplemental Fig. 1). In lieu of costly molecular methods, many of these species descriptions rely solely on a morphological framework. These analyses distinguish species from their closest congener(s), diagnose species within their local region, and leave them unassigned or ambiguously assigned to a more inclusive species-group. This is compounded by rapid species discovery which outpaces a phylogenetic understanding of this immensely successful genus.

*Cyrtodactylus* ranges from Pakistan and western India eastward to the Solomon Islands and in doing so covers an enormous expanse of ecoregions and global biodiversity hotspots^35^. Given the distributional spread across geopolitical borders, the number of researchers involved, and methods of specimen collection, it remains a challenge to keep current with the systematics of this group. Biodiversity estimates are consistently underreported for a number of countries within the range of *Cyrtodactylus*. With increased attention and sampling throughout Southeast Asia, specifically in the Indochinese, Sundaic, Philippine, Wallacean, and Papuan regions, it remains vital to maintain consistency in methods for accurate records of species diversity. Where barcoding datasets do exist for *Cyrtodactylus*, they have been created almost exclusively for species descriptions^21, 24, 25^. Often these barcoding phylogenies are carried out within the confines of a single country, such as for Laos^36^ and Vietnam^20, 37^. The complex geological histories of the regions across which *Cyrtodactylus* occurs, and the convoluted biogeographic history of the genus itself, make these ‘barcode-by-country’ reviews potentially misleading in their phylogenetic conclusions. Indeed, more inclusive molecular phylogenies are already beginning to resolve the synonymy of a number of bent-toed gecko species^38^. And while we are aware of no researchers who would agree with a geopolitically monophyletic hypothesis (clades are restricted to country borders) for *Cyrtodactylus*, ‘barcode-by-country’ reviews continue to unintentionally make just such phylogenetic assumptions.

Herein, we highlight the utility of the barcoding marker *COI* for intraspecific and shallow interspecific phylogenetic use, and encourage its use as an alternative to morphology-only systematic comparison. Additionally, we hope to draw attention to the potentially damaging practice of “barcoding-by-country,” by elucidating the fractured biogeographic history of *Cyrtodactylus* throughout the Indochinese region. We use Vietnam as an explicit example of a geopolitical boundary thought to be inhabited by three independent lineages^22^, to encourage a broader comparison of *Cyrtodactylus* in taxonomic and systematic works. Ultimately, for researchers without access to funding or sequencing facilities, DNA barcoding with the Cold Code continues to allow us all to work towards more complete sampling of *Cyrtodactylus*, providing a more accurate picture of the taxonomic and morphological diversity of this genus.

## Results

### Phylogenetic Inference using COI and ND2

**Figure 1:**
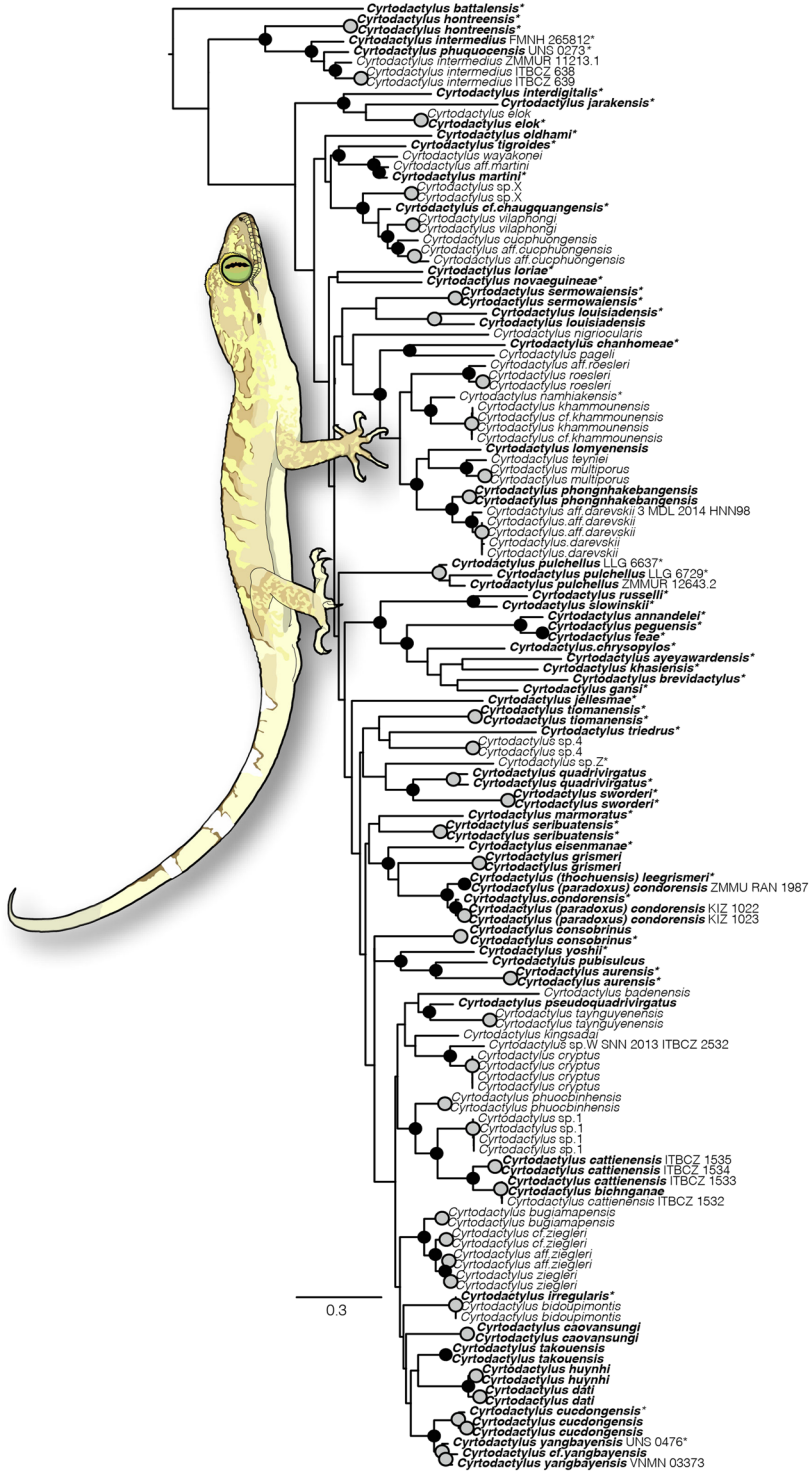
‘Fully-sampled’ maximum likelihood phylogeny of *Cyrtodactylus* as inferred from mitochondrial locus *COI*, including novel sequences contributed by this study (51) indicated by asterisks. Circles at nodes indicate BSS values of ≥70: grey indicate intraspecific sampling and black interspecific sampling. Bolded names indicate samples also included in the ‘Standardized *ND2*’ phylogeny (Fig. 2). Sample numbers are included to aid in determining relationships in cases where more than 2 samples were used for a given species, or species are reconstructed as paraphyletic. *Cyrtodactylus pubisulcus* image drawn by IGB from photograph courtesy of Ben Karin.

**Figure 2:**
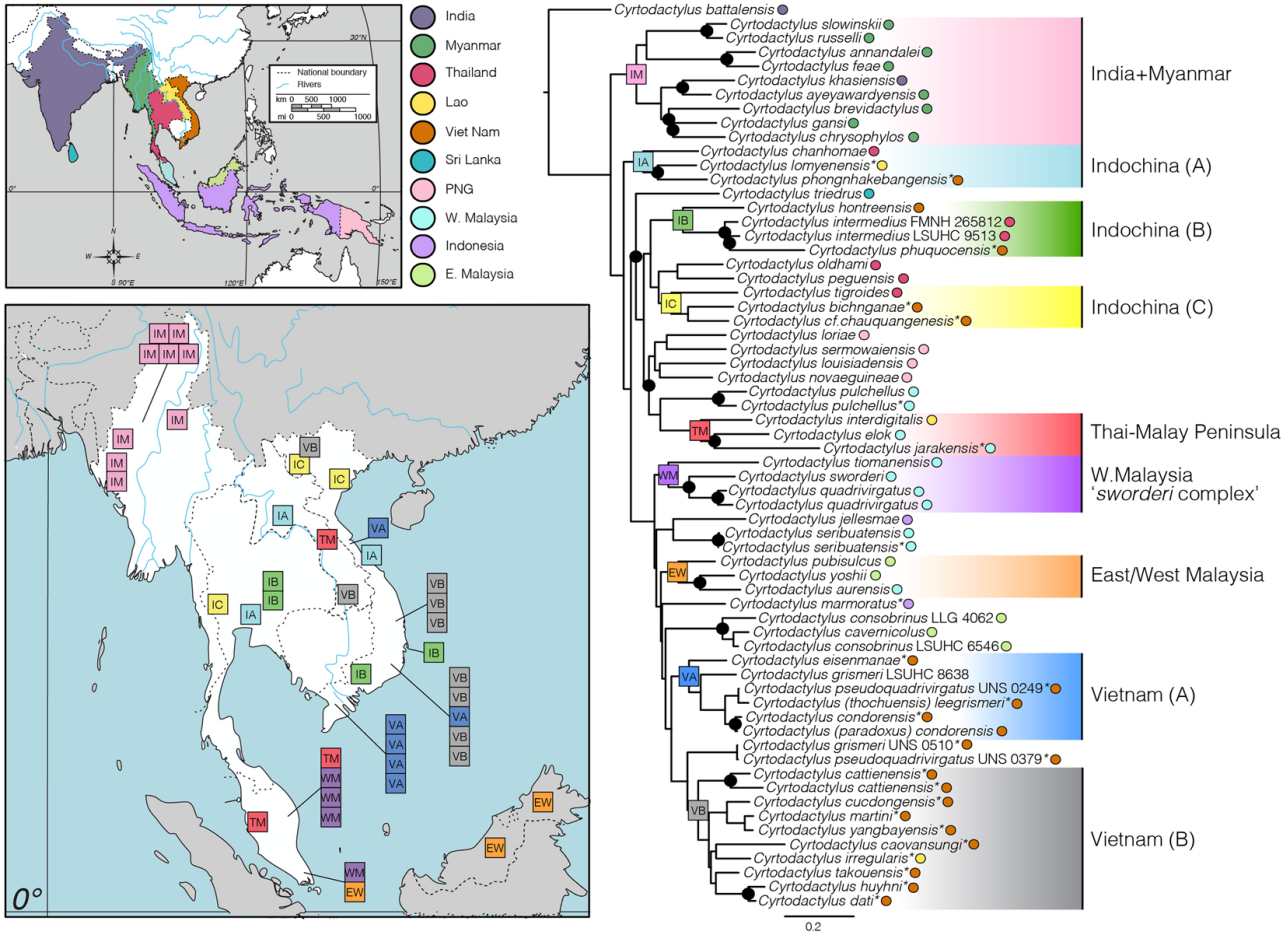
‘Standardized *ND2*’ Maximum likelihood genealogy of *ND2* including only taxa for which *COI* sequence data also exist. Circles at nodes indicate clade congruence between *ND2* and *COI* loci, with BSS values of ≥70: blue indicate species groups, black interspecific sampling. Asterisks indicate new *ND2* sequences contributed by this study. Upper map shows the geopolitical distribution of samples included in this phylogeny, and colored circles associated with tree tips correspond to this map. Lower map highlights the Indochinese region, and boxes represent generalized sampling localities of species groups (IM, IA, IB, IC, TM, WM, EW, VA, VB; denoted by blue circles at nodes). Sampled country localities indicated by colored circles at the tree tips highlight the interdigitated nature of geographic relationships within phylogenetic species groups. Maps drawn and adapted by IGB in Adobe Illustrator CS6 from public domain image provided by Wikimedia Commons (https://commons.wikimedia.org/wiki/File:Location_Map_Asia.svg).

New sequences and those acquired from GenBank included a total of 63 individuals sampled for both mitochondrial markers. In the fully sampled *COI* (Fig. 1) and the *COI*/*ND2*-standardized genealogies (Fig. 2), deeper relationships within *Cyrtodactylus* obtained very little support. However, nearly all (37/39) intraspecific relationships were strongly supported (BSS ≥ 90%). Sister-taxa relationships are also well supported (≥70%) in both full and standardized genealogies. As expected, no support existed for reciprocal monophyly of current geopolitical regions.

The genealogy based on *ND2* and standardized to our *COI* sampling strongly supported the majority of intraspecific relationships (Fig. 2). Analyses of sampling-standardized *ND2* obtained greater and more frequent support for sister-taxa relationships, as well as strong support (≥90%) at a number of deeper nodes that denoted species-groups of *Cyrtodactylus* (Fig. 2; colored boxes denote geographic region). Biogeographic matrilines returned by analysis of *ND2* were largely consistent with those presented by Wood *et al*.^22^, albeit with reduced support.

### Congruence in Mitochondrial Markers

Prior phylogenetic reconstructions (combined mitonuclear) of *Cyrtodactylus* found mtDNA matrilineal genealogies and nDNA phylogenies were largely congruent^22, 23, 32^. Matrilineal phylogeny as inferred by *ND2* has been valuable in predicting accurate phylogenetic relationships within *Cyrtodactylus* ^22^. Both *ND2* and *COI* genealogies strongly supported the monophyly of several species groups that were obtained consistently in other investigations of *Cyrtodactylus* ^23, 32, 39–41^. Exclusive of *C*. *battalensis—*the sole representative of the West Himalayan group—there was strong support (91-*ND2*/72-*COI*) for the monophyly of an India-Myanmar (IM) sister-group to the remaining species of *Cyrtodactylus*. Both genealogies supported three independent Indochinese groups: (A; IA) *C*. *chanhomae*, *C*. *lomyenensis*, and *C*. *phongnhakebangensis* (96/83); (B; IB) *C*. *hontreensis*, *C*. *intermedius*, and *C*. *phuquocensis* (98/72); and (C; IC) *C*. *tigroides*, *C*. *bichnganae*, and *C*. *cf*. *chauquangensis* (99/70). These matrilines included residents of Thailand, Laos, and Vietnam, without geopolitical monophyly. Members of the ‘*C*. *sworderi* complex’ (WM)^39, 40^ varied in support (100/65), as did an East/West Malaysian (EW) group composed of *C*. *pubisulcus*, *C*. *yoshii*, and *C*. *aurensis* (88/72). Moderate support existed for a Thai/Malay Peninsula (TM) matriline comprised of *C*. *interdigitalis*, *C*. *elok*, and *C*. *jarakensis*. Additionally, there was strong support for distinct Vietnamese groups A (VA) (100/73) and B (VB) (85/75), although no consistent support united them into a monophyletic group (55/40). Indochinese species from Vietnam, Thailand, and Laos were assigned to multiple clades (5, 3, and 3, respectively), which were strongly supported across both molecular datasets.

## Discussion

As in any field, assessing the appropriateness of the data to resolve the question of interest is paramount. In molecular systematics studies, this means addressing the ability of the data to provide phylogenetic information at the evolutionary depth or depths of interest. DNA barcoding has been lauded as a way to cheaply and rapidly include molecular data into species descriptions and phylogenetic studies. However, the evolutionary scale of the group of interest often resides outside the limits of barcoding’s phylogenetic reconstruction abilities. We find that *COI* alone can not replace phylogenetic assessment by multilocus mitonuclear study, nor does it resolve relationships as accurately as another, single mitochondrial locus (*ND2*). What it does provide however, is valuable information for shallow scale interspecific and intraspecific systematics, which are invaluable to species discovery.

When viewed in its entirety, instead of by geopolitical boundaries, *Cyrtodactylus* show a general West to East biogeographic trend^22^. A number of eastward dispersals of Indochinese origin into the Sundaic, Wallacean, Papuan, and Philippine regions punctuate this overall pattern^22^. These dispersal events account for the distribution of geographically proximate species interspersed across the tree of *Cyrtodactylus*. This is particularly relevant to the appropriate differential diagnosis of novel taxa. Some groups of *Cyrtodactylus* are easy to identify morphologically from geographic congeners, such as ground-dwelling members of the subgenus *Geckoella* from India and Sri Lanka^23^, Papuan giants^42^, and Sundaic dwarves^43^. In contrast, however, Vietnamese bent-toed geckos represent a prime example of a morphologically conservative body plan involving multiple species groups. Our trees depict five well supported matrilines of Vietnamese *Cyrtodactylus* (Fig. 2; orange circles) interspersed with inhabitants of other Indochinese and Sundaic nations. This convoluted biogeographic history highlights the necessity of molecular and morphological comparison against closest phylogenetic and not solely political congeners.

Barcoding initiatives across the tree of life largely coincide with an interest in species discovery and delimitation. At least 12 species of *Cyrtodactylus* have been described since 2012 using a combination of morphological means and barcoding data. However, during that same period, several other species have been described based solely on morphological assessments^26, 44–48^. Prior to the initiation of DNA barcoding and Cold Code, the inclusion of molecular data into species descriptions was time-intensive, costly, and limited significantly by access to sequencing resources. The advent of Cold Code and the introduction of subsidized genetic barcoding makes it possible to include molecular results in species descriptions. Notwithstanding, barcoding is not the ultimate phylogenetic tool because it offers a matrilineal perspective on the history of species only, and the rapid evolution of barcoding genes often precludes the resolution of deep relationships.

DNA barcoding in other taxa has, unfortunately, unsuccessfully resolved interspecific relationships, identified independently evolving lineages, and, worse, misidentified interspecific relationships as a result of mitogenome introgression^13–16^. Our analyses address the use of genetic barcoding as a method for inferring historical associations among species of *Cyrtodactylus* via direct comparison with another popular mitochondrial marker *ND2*. Prior to the implementation of Cold Code, alternative mitochondrial markers such as *ND2*, *16S*, and *cytb* have been used more frequently as markers for identifying independently evolving units for taxonomic description. However, as DNA barcoding has become more popular, *COI* has supplanted alternatives due to its near-universal applicability. *COI* also is the dominant marker for describing and inferring relationships between novel taxa within this genus. As a result, many species of *Cyrtodactylus* have been described using morphology in combination with either *COI* or *ND2*, but rarely both molecular markers. Here, our assessment adds 46 additional samples to allow for direct comparison of both loci, to assess the value of *COI* as a phylogenetic tool in *Cyrtodactylus*.

Neither *COI* nor *ND2* successfully resolve deeper relationships within *Cyrtodactylus* with much support. This result likely owes to the phylogenetic depth, i.e. age of the genus, and the limitations of employing a single locus. Notwithstanding, the matrilineal phylogeny as inferred using *ND2* is largely concordant with the nuclear DNA phylogeny of Wood *et al*.^22^. Moderate to strong levels of support for a series of species-groups in Fig. 2 highlights the value of *COI* at resolving shallow interspecific relationships that are consistent with those of *ND2*. The smaller fragment of *COI* (658 bp) and slower mutational rate when compared to *ND2* (1047 bp + 400 bp of tRNAs) hamper phylogenetic inference beyond close relationships (Fig. 1). As an identifier of species groups, *COI* performs moderately well by providing support for 9 of 12 matrilines obtained with strong support by analysis of *ND2*.

DNA barcoding has been used most frequently in *Cyrtodactylus* as a method for describing and inferring relationships between novel taxa. Most of these investigations have used *COI* exclusively, and because of this, *COI* and *ND2* datasets are largely non-overlapping. The standardizing of datasets across mitochondrial loci serves to evaluate the phylogenetic utility of *COI* as a tool for genealogical inference relative to *ND2*. Ultimately, many sister-taxa and some higher level relationships as suggested by our fully sampled *COI* tree cannot be tested against *ND2* due to sampling. While *COI* plays a valuable role in species discovery and as a tool for informing other comparative methods (morphology, ecology, biogeography), we also recognize its shortcomings. When possible, we encourage the use of additional molecular markers (*ND2*, *RAG1*, *PDC*, *MXRA5*) for inferring relationships within this ultra-diverse genus. Ultimately, confident resolution may require massive amounts of data that next generation genomic sequencing yields, either complete mitogenomes, or SNPs from nuclear DNA. In addition to Cold Code-funded barcode sequencing, we encourage potential descriptors of new species of *Cyrtodactylus* to contact IGB and AMB regarding the possibility of additional molecular sequencing.

When used as the sole molecular marker for phylogenetic inference of a group of any considerable depth, or as an intraspecific marker for tracking matrilineal history, *COI* is unlikely to provide the resolution desired to confidently support or refute hypotheses. When appropriately used as part of a pluralistic methodology, however, DNA barcoding may prove extremely useful. Prior molecular assessment or “genetic screening” can help accurately place a novel species into a species group for the most useful morphological comparison. While it is important to diagnose new taxa in reference to geographic congeners, it is also necessary to distinguish it from its closest evolutionary congeners, to help develop a more complete image of its history. The high expense of DNA sequencers and satellite equipment and time-intensive methods continue to impede the inclusion of genetic data in species’ descriptions. In response, Cold Code provides cost-free sequencing of the DNA barcoding locus *COI* for up to 10 individuals of any species.

## Materials and Methods

### Ethics

Field and laboratory experimental protocol for NSF subaward 13–0632 and DEB 0844532 were approved by Villanova University IACUC (approval: 16-14 and 11-04 respectively). *Cyrtodactylus* samples were collected in compliance with permits to NVT at the Institute of Tropical Biology, under the Vietnam Academy of Science and Technology, following guidelines of the Institutional Animal Care and Use Committee (IACUC).

### Taxon Sampling and Molecular Methods

New sampling for this project was built upon molecular datasets assembled for investigations into inter- and intraspecific relationships within *Cyrtodactylus* ^21–25, 36, 37, 39–41, 49^. A large number of sequences were acquired from GenBank, but to this growing dataset we have sequenced 51 additional samples for *COI*, and a further 25 samples sequenced for the mitochondrial locus *ND2*. Due to its comparatively fast mutation rate, length, history in the literature, and ease of amplification, *ND2* has been used consistently in studies of squamate phylogenetics (>20,900 GenBank records), and as the primary locus for the systematics of *Cyrtodactylus* (>900 GenBank records). For these reasons we have chosen to compare *COI* directly to *ND2*, for use in bent-toed gecko phylogenetics. All samples are accompanied by locality data, voucher information, and GenBank accession numbers, recorded in Table 1.

**Table 1:** List of samples used in this study with appropriate voucher (museum or field) numbers, locality data, and GenBank accession numbers.

Genus & species	Collection #	Locality	Country	Genbank #	
				COI	ND2
*Cyrtodactylus aff*. *cucphuongensis*	MDL 2014 AT 2013 2	NA	Vietnam	KJ817428	—
*Cyrtodactylus puhuensis*	SNN 2013a KIZ 11665	Houphan Province	Laos	KF929529	—
*Cyrtodactylus aff*. *darevskii*	3 MDL 2014 HNN 98	Khammouane Province	Laos	KJ817429	—
*Cyrtodactylus aff*. *darevskii*	SNN 2013d ZISPFN 185	Na Hom Village, Khammouan Province	Laos	KF929542	—
*Cyrtodactylus aff*. *darevskii*	SNN 2013d ZISPFN 186	Na Hom Village, Khammouan Province	Laos	KF929543	—
*Cyrtodactylus aff*. *martini*	SNN 2013c KIZ 2011.03	Xishuangbanna, Yunnan Province	China	KF929537	—
*Cyrtodactylus aff*. *roesleri*	4 MDL 2014 HNN 68	Khammouane Province	Laos	KJ817437	—
*Cyrtodactylus aff*. *ziegleri*	SNN 2013 VNMN 2014	Na Nung, Dak Nong Province	Vietnam	KF169975	—
*Cyrtodactylus aff*. *ziegleri*	SNN 2013 VNMN 2015	Na Nung, Dak Nong Province	Vietnam	KF169976	—
*Cyrtodactylus annadalei*	CAS 215722	Alaung Daw Kathapa NP	Myanmar	MF169899	JX440524
*Cyrtodactylus aurensis*	LSUHC 7286	Pulau Aur, Johor	W. Malaysia	MF169900	JX440525
*Cyrtodactylus aurensis*	LSUHC 7300	Pulau Aur, Johor	W. Malaysia	MF169901	—
*Cyrtodactylus ayeyawardensis*	CAS 216459	Than Dawe District, Rakhine State	Myanmar	MF169902	JX440526
*Cyrtodactylus badenensis*	KIZ 13689	Mt. Ba Den, Tay Ninh Province	Vietnam	KF929505	—
*Cyrtodactylus battalensis*	PMNH 2301	Battagram City, NWFP	Pakistan	MF169903	KC152035
*Cyrtodactylus bichnganae*	UNS 0473	Son La Urban, Son La Province	Vietnam	MF169904	MF169953
*Cyrtodactylus bidoupimontis*	ITBCZ 1536	Bi Doup, Nui Ba NP, Lam Dong Province	Vietnam	KF169958	—
*Cyrtodactylus bidoupimontis*	ITBCZ 1537	Bi Doup, Nui Ba NP, Lam Dong Province	Vietnam	KF169959	—
*Cyrtodactylus brevidactylus*	CAS 214104	Popa Mountain Park, Mandalay Division	Myanmar	MF169905	JX440527
*Cyrtodactylus bugiamapensis*	ITBCZ 1562	Bu Gia Map NP	Vietnam	KF169961	—
*Cyrtodactylus bugiamapensis*	KIZ 45	Bu Gia Map NP	Vietnam	KF169965	—
*Cyrtodactylus caovansungi*	ITBCZ 2305; UNS 0304	Nui Chua NP, Ninh Thuan Province	Vietnam	—	MF169954
*Cyrtodactylus caovansungi*	ITBCZ 1113	Nui Chua NP, Ninh Thuan Province	Vietnam	KF219680	—
*Cyrtodactylus caovansungi*	ITBCZ 932	Nui Chua NP, Ninh Thuan Province	Vietnam	KF219679	—
*Cyrtodactylus cattienensis*	UNS 0368	Ma Da SFE, Dong Nai Province	Vietnam	—	MF169955
*Cyrtodactylus cattienensis*	UNS 0389	Ma Da SFE, Dong Nai Province	Vietnam	—	MF169956
*Cyrtodactylus cattienensis*	ITBCZ 1532	Cat Tien NP	Vietnam	KF169956	—
*Cyrtodactylus cattienensis*	ITBCZ 1533	Cat Tien NP	Vietnam	KF169957	—
*Cyrtodactylus cattienensis*	ITBCZ 1534	Cat Tien NP	Vietnam	KF929506	—
*Cyrtodactylus cattienensis*	ITBCZ 1535	Cat Tien NP	Vietnam	KF929507	—
*Cyrtodactylus cavernicolus*	LSUHC 4056	Niah Cave, Sarawak	E. Malaysia	—	JX440528
*Cyrtodactylus cavernicolus*	LLG 4055	Niah Cave, Sarawak	E. Malaysia	MF169906	—
*Cyrtodactylus cf*. *chaquangensis*	UNS 0505	Chau Quang Commune, Nghe An Province	Vietnam	MF169907	MF169957
*Cyrtodactylus cf*. *khammounensis*	SNN 2013e ZISPFN 191	Na Hom Village, Khammouan Province	Laos	KF169958	—
*Cyrtodactylus cf*. *khammounensis*	SNN 2013e ZISPFN 192	Na Hom Village, Khammouan Province	Laos	KF169959	—
*Cyrtodactylus cf*. *yangbayensis*	RuHF ZMMU R 13090.1	Ba Ho cascade, Khanh Hoa Province	Vietnam	KC016081	—
*Cyrtodactylus cf*. *ziegleri*	ITBCZ 2051; UNS 5006	Chu Yang Sin NP, Dak Lak Province	Vietnam	KF169946	—
*Cyrtodactylus cf*. *ziegleri*	ITBCZ 2052; UNS 5007	Chu Yang Sin NP, Dak Lak Province	Vietnam	KF169945	—
*Cyrtodactylus chanhomae*	CUM Z 2003.62	Thep Nimit Cave, Saraburi Province	Thailand	MF169908	JX440529
*Cyrtodactylus chrysophylos*	CAS 226141	Panlaung-Pyadalin Cave, Shan State	Myanmar	MF169909	JX440530
*Cyrtodactylus condorensis*	ITBCZ 2231; UNS 0431	Con Dao NP, Ba Ria-Vung Tau Province	Vietnam	MF169910	MF169958
*Cyrtodactylus consobrinus*	LSUHC 4062	Niah Cave, Sarawak	E. Malaysia	—	EU268349
*Cyrtodactylus consobrinus*	LSUHC 6546	Selangor	W. Malaysia	MF169911	JX440532
*Cyrtodactylus consobrinus*	ZMMUR 12644.1	“without precise locality”	Malaysia	HQ967204	—
*Cyrtodactylus cryptus*	PNKB 1	Phong Nha-Ke Bang NP	Vietnam	KF169969	—
*Cyrtodactylus cryptus*	PNKB 2	Phong Nha-Ke Bang NP	Vietnam	KF169970	—
*Cyrtodactylus cryptus*	PNKB 3	Phong Nha-Ke Bang NP	Vietnam	KF169971	—
*Cyrtodactylus cryptus*	PNKB 4	Phong Nha-Ke Bang NP	Vietnam	KF169972	—
*Cyrtodactylus cucdongensis*	ITBCZ 2344; UNS 0544	Hon Heo Mountain, Khanh Hoa Province	Vietnam	Awaiting accession	MF169959
*Cyrtodactylus cucdongensis*	VNMN A 2013 18	Cuc Dong Cape, Khanh Hoa Province	Vietnam	KJ403845	—
*Cyrtodactylus cucdongensis*	ZFMK 95513	Cuc Dong Cape, Khanh Hoa Province	Vietnam	KJ403847	—
*Cyrtodactylus cucphuongensis*	ITBCZ 2206; UNS 0406	Cuc Phuong NP, Ninh Binh Province	Vietnam	MF169912	—
*Cyrtodactylus darevskii*	RN 2012 ZISP FN 187	Na Home, Boulapha, Khammouane Province	Laos	HQ967223	—
*Cyrtodactylus darevskii*	RN 2012 ZISP FN 188	Na Home, Boulapha, Khammouane Province	Laos	HQ967225	—
*Cyrtodactylus dati*	ITBCZ 2343; UNS 0543	Bu Dop, Binh Phuoc Province	Vietnam	—	MF169960
*Cyrtodactylus dati*	ITBCZ 2537	Bu Dop, Binh Phuoc Province	Vietnam	KF929508	—
*Cyrtodactylus dati*	ITBCZ 2538	Bu Dop, Binh Phuoc Province	Vietnam	KF929509	—
*Cyrtodactylus eisenmanae*	LSUHC 8598	Hon Son Island, Kien Giang Province	Vietnam	—	JX440534
*Cyrtodactylus eisenmanae*	UNS 0479	Hon Son Island, Kien Giang Province	Vietnam	MF169913	MF169961
*Cyrtodactylus elok*	LSUHC 6471	Fraser’s Hill, Pahang	W. Malaysia	—	JQ889180
*Cyrtodactylus elok*	JB 14	Captive	NA	MF169914	—
*Cyrtodactylus elok*	ZMMU RAN 1991	“without precise locality”	Malaysia	HM888478	—
*Cyrtodactylus feae*	USNM 559805	Popa Mountain Park, Mandalay Division	Myanmar	MF169915	JX440536
*Cyrtodactylus gansi*	CAS 222412	Min Dat District, Chin State	Myanmar	MF169916	JX440537
*Cyrtodactylus grismeri*	LSUHC 8638	Tuc Dup Hill, An Giang Province	Vietnam	—	JX440538
*Cyrtodactylus grismeri*	UNS 0510	Tuc Dup Hill, An Giang Province	Vietnam	—	MF169962
*Cyrtodactylus grismeri*	ITBCZ 683	Mt. Tuc Dup, An Giang Province	Vietnam	KF929512	—
*Cyrtodactylus grismeri*	ITBCZ 684	Mt. Tuc Dup, An Giang Province	Vietnam	KF929513	—
*Cyrtodactylus hontreensis*	LSUHC 8583	Hon Tre Island, Kien Giang Province	Vietnam	MF169917	JX440539
*Cyrtodactylus huynhi*	UNS 0413	Chua Chan Mountain, Dong Nai Province	Vietnam	—	MF169963
*Cyrtodactylus huynhi*	ITBCZ 511	Mt. Chua Chan, Dong Nai Province	Vietnam	KF169947	—
*Cyrtodactylus interdigitalis*	FMNH 255454	Nakai District, Khammouan Province	Lao PDR	MF169919	JQ889181
*Cyrtodactylus intermedius*	FMNH 265812	Muang Sa Kaeo, Sa Kaeo	Thailand	MF169920	JQ889182
*Cyrtodactylus intermedius*	LSUHC 9513	Khao Khitchakut, Chantaburi Province	Thailand	—	JX519469
*Cyrtodactylus intermedius*	ITBCZ 638	Mt. Nui Cam, An Giang Province	Vietnam	KF929521	—
*Cyrtodactylus intermedius*	ITBCZ 639	Mt. Nui Cam, An Giang Province	Vietnam	KF929522	—
*Cyrtodactylus intermedius*	ZMMU R 11213 1	Phnom Bakor NP	Cambodia	KC016076	—
*Cyrtodactylus irregularis*	FMNH 258697	Pakxong District, Champasak Province	Lao PDR	—	JX440540
*Cyrtodactylus irregularis*	UNS 0269	Bi Doup, Nui Ba NP, Lam Dong Province	Vietnam	MF169921	MF169964
*Cyrtodactylus jarakensis*	LSUHC 8990	Pulau Jarak, Perak	W. Malaysia	MF169922	MF169965
*Cyrtodactylus jellesmae*	MVZ 239337	Propinsi Sulawesi Selatan, Sulawesi	Indonesia	MF169923	JX440542
*Cyrtodactylus khammounensis*	RN 2012 ZISP FN 191	Na Hom Village, Khammouan Province	Laos	HM888467	—
*Cyrtodactylus khammounensis*	RN 2012 ZISP FN 192	Na Hom Village, Khammouan Province	Laos	HM888468	—
*Cyrtodactylus khasiensis*	MFA 50083	Kaziranga, Assam	India	MF169924	JX440543
*Cyrtodactylus kingsadai*	IEBRA 2013 3	Dai Lanh, Phu Yen Province	Vietnam	KF188432	—
*Cyrtodactylus lomyenensis*	UNS 0534	Lom Yen Cave, Khammouane Province	Laos	—	MF169966
*Cyrtodactylus lomyenensis*	IEBR KM 2012.54	Lom Yen, Gnommalath, Khammouane Province	Laos	KP199942	—
*Cyrtodactylus loriae*	FK 7709	Mt. Simpson, Milne Bay Province	Papua New Guinea	MF169925	EU268350
*Cyrtodactylus louisiadensis*	NA	Sudest Island	Papua New Guinea	—	HQ401190
*Cyrtodactylus louisiadensis*	BPBM 15434	Mt. Pekopekowana, Milne Bay Province	Papua New Guinea	MF169926	—
*Cyrtodactylus louisiadensis*	BPBM 18654	Apele, Morobe Province	Papua New Guinea	MF169927	—
*Cyrtodactylus marmoratus*	ABTC 48075	Java	Indonesia	—	GQ257747
*Cyrtodactylus marmoratus*	JAM 2242	NA	NA	MF169928	MF169967
*Cyrtodactylus martini*	UNS 0471	Lai Chau Province	Vietnam	MF169929	MF169968
*Cyrtodactylus multiporus*	RN 2012 ZMMU RAN 1996 2	Na Hom Village, Khammouan Province	Laos	HQ967193	—
*Cyrtodactylus multiporus*	RN 2012 ZMMU RAN 1998	Na Hom Village, Khammouan Province	Laos	HQ543943	—
*Cyrtodactylus namhiakensis*	UNS 0529	Nam Hiak Cave, Khammouane Province	Vietnam	MF169930	—
*Cyrtodactylus nigriocularis*	VNMN 2187	Mt. Ba Den, Tay Ninh Province	Vietnam	KF929523	—
*Cyrtodactylus novaeguineae*	BPM 23316	Toricelli Mountains, West Sepik Province	Papua New Guinea	—	JX440547
*Cyrtodactylus novaeguineae*	BMBM 18655	Mt. Shungoi, Morobe Province	Papua New Guinea	MF169931	—
*Cyrtodactylus oldhami*	JB 126	captive	NA	MF169932	JX440548
*Cyrtodactylus pageli*	ZFMK 91827	Vientiane Province	Laos	KJ817431	—
*Cyrtodactylus* (*paradoxus*) *condorensis*	LSUHC 8672	Hon Nghe Island	Vietnam	—	JX440549
*Cyrtodactylus* (*paradoxus*) *condorensis*	KIZ 1022	Hon Chong, Kien Giang Province	Vietnam	KF929524	—
*Cyrtodactylus* (*paradoxus*) *condorensis*	KIZ 1023	Hon Chong, Kien Giang Province	Vietnam	KF929525	—
*Cyrtodactylus* (*paradoxus*) *condorensis*	ZMMU RAN 1987	Koh Tang Island	Cambodia	HM888464	—
*Cyrtodactylus peguensis*	CUM Z R2005.07.30.54	Khao Luang NP	Thailand	—	GU550727
*Cyrtodactylus peguensis*	CAS 214029	Popa Mountain Park, Mandalay Division	Myanmar	MF169933	—
*Cyrtodactylus phongnhakebangensis*	UNS 0347	Phong Nha-Ke Bang NP, Quang Binh Province	Vietnam	—	MF169970
*Cyrtodactylus phongnhakebangensis*	PNKN 2011.30	Phong Nha-Ke Bang NP, Quang Binh Province	Vietnam	KF929526	—
*Cyrtodactylus phongnhakebangensis*	PNKN 2011.32	Phong Nha-Ke Bang NP, Quang Binh Province	Vietnam	KF929527	—
*Cyrtodactylus phuquocensis*	UNS 0273	Phu Quoc NP, Kien Giang Province	Vietnam	MF169934	MF169971
*Cyrtodactylus pseudoquadrivirgatus*	UNS 0249	Ba Na NR, Da Nang City	Vietnam	—	MF169972
*Cyrtodactylus pseudoquadrivirgatus*	UNS 0379	Son Tra NR, Da Nang City	Vietnam	—	MF169973
*Cyrtodactylus pseudoquadrivirgatus*	ITBCZ 30001	A Luoi, Hue Province	Vietnam	KF169963	—
*Cyrtodactylus pubisulcus*	LSUHC 4069	Niah Cave, Sarawak	E. Malaysia	—	JX4405510
*Cyrtodactylus pubisulcus*	ZMMUR 13091.3	near Tondong, Sarawak	E. Malaysia	HQ967199	—
*Cyrtodactylus pulchellus*	LSUHC 6637	Genting Highlands, Selangor	NA	MF169935	—
*Cyrtodactylus pulchellus*	LSUHC 6729	Moongate Trail, Pulau Pinang	W. Malaysia	MF169936	MF169974
*Cyrtodactylus pulchellus*	ZMMU R 12643.2	“without precise locality”	Malaysia	HQ967201	—
*Cyrtodactylus quadrivirgatus*	LSUHC 4813	Pulau Tioman, Pahang	W. Malaysia	—	JX440553
*Cyrtodactylus quadrivirgatus*	LSUHC 9869	Bukit Larut, Perak	W. Malaysia	—	JQ889252
*Cyrtodactylus quadrivirgatus*	JB 78	Captive	NA	MF169937	—
*Cyrtodactylus quadrivirgatus*	ZMMUR AN 1990	“without precise locality”	Malaysia	HM888466	—
*Cyrtodactylus roesleri*	PNKB 20111	Phong Nha-Ke Bang NP	Vietnam	KF929530	—
*Cyrtodactylus roesleri*	PNKB 20113	Phong Nha-Ke Bang NP	Vietnam	KF929531	—
*Cyrtodactylus russelli*	CAS 226137	Htamanthi Wildlife Sanctuary, Sagaing Division	Myanmar	MF169938	JX440555
*Cyrtodactylus seribuatensis*	LSUHC 6348	Pulau Mentigi, Johor	W. Malaysia	MF169939	JX440557
*Cyrtodactylus seribuatensis*	LSUHC 6349	Pulau Mentigi, Johor	W. Malaysia	MF169940	MF169976
*Cyrtodactylus sermowaiensis*	BPM 23317	Toricelli Mountains, West Sepik Province	Papua New Guinea	—	JX440558
*Cyrtodactylus sermowaiensis*	BMBM 23317	Toricelli Mountains, West Sepik Province	Papua New Guinea	MF169941	—
*Cyrtodactylus sermowaiensis*	BPBM 23320	Toricelli Mountains, West Sepik Province	Papua New Guinea	MF169942	—
*Cyrtodactylus slowinskii*	CAS 210205	Alaung Daw Kathapa NP	Myanmar	MF169943	JX440559
*Cyrtodactylus sp*. *1*	RuHF ZMMU R 11503.2	Mt. Nui Chua NP, Ninh Thuan Province	Vietnam	KC016080	—
*Cyrtodactylus sp*. *1*	SNN 2013 ITBCZ 1150	Mt. Nui Chua NP, Ninh Thuan Province	Vietnam	KF929540	—
*Cyrtodactylus sp*. *1*	SNN 2013 ITBCZ 965	Mt. Nui Chua NP, Ninh Thuan Province	Vietnam	KF929538	—
*Cyrtodactylus sp*. *1*	SNN 2013b ITBCZ 1117	Mt. Nui Chua NP, Ninh Thuan Province	Vietnam	KF929539	
*Cyrtodactylus sp*. *W*	SNN 2013 ITBCZ 2532	Ba Na Resort, Da Nang City	Vietnam	KF169962	—
*Cyrtodactylus phuocbinhensis*	SNN 2013 ITBCZ 1518	Phuoc Binh NP	Vietnam	KF169953	—
*Cyrtodactylus phuocbinhensis*	SNN 2013 ITBCZ 1529	Phuoc Binh NP	Vietnam	KF169954	—
*Cyrtodactylus taynguyenensis*	SNN 2013 ROM 32119	Krongpa Village, Gia Lai Province	Vietnam	KF169978	—
*Cyrtodactylus taynguyenensis*	SNN 2013 ROM 32120	Krongpa Village, Gia Lai Province	Vietnam	KF169979	—
*Cyrtodactylus sp*. *4*	RuHF ZMMU RAN 1994	NA	NA	KC016078	—
*Cyrtodactylus sp*. *4*	RuHF ZMMU RAN 1995	NA	NA	KC016079	—
*Cyrtodactylus sp*. *X*	MDL 2014 LPB 62	Luang Prabang Province	Laos	KJ817432	—
*Cyrtodactylus sp*. *X*	MDL 2014 LPB 63	Luang Prabang Province	Laos	KJ817433	—
*Cyrtodactylus sp*. *Z*	ENS 7764	Sumatra	Indonesia	MF169944	—
*Cyrtodactylus sworderi*	LSUHC 7685	Endau-Rompin, Johor	W. Malaysia	MF169945	JQ889189
*Cyrtodactylus sworderi*	LSUHC 7700	Endau-Rompin, Johor	W. Malaysia	MF169946	—
*Cyrtodactylus takouensis*	UNS 0486	Ta Kou NR, Binh Thuan Province	Vietnam	—	MF169978
*Cyrtodactylus takouensis*	ITBCZ 2527	Ta Kou NR, Binh Thuan Province	Vietnam	KF929533	—
*Cyrtodactylus takouensis*	ITBCZ 2528	Ta Kou NR, Binh Thuan Province	Vietnam	KF929534	—
*Cyrtodactylus teyniei*	KM 2012.77	Khammouane Province	Laos	KP199945	—
*Cyrtodactylus* (*thochuensis*) *leegrismeri*	UNS 0498	Tho Chu Island, Kien Giang Province	Vietnam	MF169947	MF169979
*Cyrtodactylus tigroides*	IRSNB 2380	Sai-Yok District, Kanchanaburi Province	Thailand	MF169948	JX440562
*Cyrtodactylus tiomanensis*	LSUHC 6251	Pulau Tioman, Pahan	W. Malaysia	MF169949	JX440563
*Cyrtodactylus tiomanensis*	LSUHC 6268	Pulau Tioman, Pahan	W. Malaysia	MF169950	—
*Cyrtodactylus triedrus*	Anslem de Silva 35 A	Yakkunehela	Sri Lanka	MF169951	JX440522
*Cyrtodactylus vilaphongi*	IEBRA 2013 103	Luang Prabang Province	Laos	KJ817435	—
*Cyrtodactylus vilaphongi*	NUOL R 2013 5	Luang Prabang Province	Laos	KJ817434	—
*Cyrtodactylus wayakonei*	ZFMK 91016	Luang Nam Tha Province	Laos	KJ817438	—
*Cyrtodactylus yangbayensis*	UNS 0407	Hon Ba NR, Khanh Hoa Province	Vietnam	—	MF169980
*Cyrtodactylus yangbayensis*	UNS 0476	Yang Bay Waterfall, Khanh Hoa Province	Vietnam	MF169952	—
*Cyrtodactylus yoshii*	ZRC 2.4851	Poring Hot Spring, Sabah	E. Malaysia	Awaiting accession	JX440565
*Cyrtodactylus ziegleri*	ZMMU R 13116 3	NA	NA	HQ967210	—
*Cyrtodactylus ziegleri*	ZMMU R 13116.4	NA	NA	HQ967211	—

*Abbreviations*: Eric N Smith, University of Texas, Arlington, USA (ENS); Kunming Institute of Zoology, China (KIZ); California Academy of Sciences, USA (CAS); La Sierra University Herpetological Collection, USA (LSUHC); L. Lee Grismer field series (LLG); United States National Museum, USA (UNS); Institute of Tropical Biology Zoological Collection, Vietnam (ITBCZ); Pakistan Museum of Natural History Museum, Pakistan (PMNH); Zoological Institute, St. Petersburg (ZISPFN); Chulalongkorn University Museum of Zoology, Thailand (CUMZ); Zoological Museum Moscow State University, Russia (ZMMUR); Phong Nha-Ke Bang, Vietnam (PNKB); Zoologisches Forschungsmuseum Alexander Koenig, Germany (ZFMK); Jon Boone captive series (JB); Field Museum of Natural History, USA (FMNH); Museum of Vertebrate Zoology, University of California, Berkeley, USA (MVZ); Institute of Ecology and Biological Resources, Vietnam (IEBRA); M. Firoz Ahmed field series (MFA); Fred Kraus field series (FK); Australian Biological Tissue Collection, Australia (ABTC); Bernice P. Bishop Museum (BPBM); Royal Ontario Museum, Canada (ROM); Institute des Sciences Naturelles du Belgique, Belgium (IRSNB); National University of Laos, Laos (NUOL); Zoological Research Collection, Raffles Museum of Biodiversity, National University of Singapore (ZRC); Jimmy A. McGuire (JAM).

After extracting genomic DNA from liver, heart, or tail tissue preserved in 95–100% ethanol via Qiagen DNeasy Blood and Tissue kits (Qiagen), isolated DNA was quantified using a NanoDrop spectrophotometer (Thermo Scientific). Samples for *COI* amplification and sequencing were sent to South China DNA Barcoding Center at the Kunming Institute of Zoology. *ND2* samples were amplified via polymerase chain reaction using standard primers and protocols^22^. All sequences were assembled, edited, and aligned in Geneious v.7, and protein-coding regions were translated to amino acid sequences to maintain proper reading frames and avoid premature stop codons. tRNA secondary structure was addressed and adjusted by eye for consistency. Final *COI* and *ND2* alignments stretched 677 and 1,512 bp, respectively.

### Phylogenetic Analyses

Datasets of mitochondrial loci *COI* and *ND2* were analyzed independently via the maximum likelihood (ML) framework for phylogenetic inference. The alignments of both genes were standardized to include the same species and wherever possible, the same specimens, to allow for direct comparison of results. An additional *COI* alignment of two samples per species for all available species (GenBank accession numbers of some recently described species remain unavailable) were combined to create a matrilineal genealogy representing all currently barcoded *Cyrtodactylus*.

We used the Akaike Information Criterion (AIC) in PartitionFinder^50^ to establish the most accurate models of evolution based on locus and codon position, specific to our analytical program (RAxML). ML analyses were carried out in RAxML 8.0^51^ via the CIPRES supercomputing portal^52^. *COI* was analyzed as a single locus, and *ND2* was partitioned into the protein coding region and tRNAs. We employed the GTR+I+Γ model of evolution, and ran the program for 100 independent tree searches to find the best topology, and 5000 bootstrap replicates to retrieve topological support values.

### Accession Codes (Data Availability)

All accession numbers are included in Table 1, except where pending acceptance to GenBank (noted as ‘Awaiting accession’).

## Electronic supplementary material

Supplementary Figure 1
