# Supplementary material for: Barcoding utility in a mega-diverse, cross-continental genus: keeping pace with Cyrtodactylus geckos
Source: Sci Rep. 2017 Jul 17;7:5592. doi: 10.1038/s41598-017-05261-9 (PMC5514027; doi:10.1038/s41598-017-05261-9)
Supplement: Supplementary file 1 — Supplementary Figure 1 [file 41598_2017_5261_MOESM1_ESM.pdf]

Barcoding utility in a mega-diverse, cross-continental genus: keeping pace with *Cyrtodactylus* geckos

Ian G. Brennan [a*], Aaron M. Bauer [a], Ngo Van Tri [b], Yun-yu Wang [c,d], Wen-zhi Wang [c,d], Ya-Ping Zhang [c,e], Robert W. Murphy [c, f]

[a] Division of Ecology & Evolution, Research School of Biology, The Australian National University, Canberra, ACT 2602, Australia

[b] Department of Environmental Management and Technology, Institute of Tropical Biology, Vietnamese Academy of Sciences and Technology, 85 Tran Quoc Toan Street, District 3, Ho Chi Minh City, Vietnam

[c] State Key Laboratory of Genetic Resources and Evolution State, and Yunnan Laboratory of Molecular Biology of Domestic Animals, Kunming Institute of Zoology, Chinese Academy of Sciences, Kunming 650223, China

[d] South China DNA Barcoding Center, Kunming Institute of Zoology, Chinese Academy of Sciences, Kunming 650223, China

[e] Laboratory for Conservation and Utilization of Bio-resource and Key Laboratory for Microbial Resources of the Ministry of Education, Yunnan University, 2 Cuihu N. Rd., Kunming 650091, China

[f] Centre for Biodiversity and Conservation Biology, Royal Ontario Museum, 100 Queen's Park, Toronto, Canada, M5S 2C6

*Corresponding author email address: Ian.Brennan@anu.edu.au

Running header: DNA barcoding in *Cyrtodactylus* geckos

Submission discipline: Phylogeny

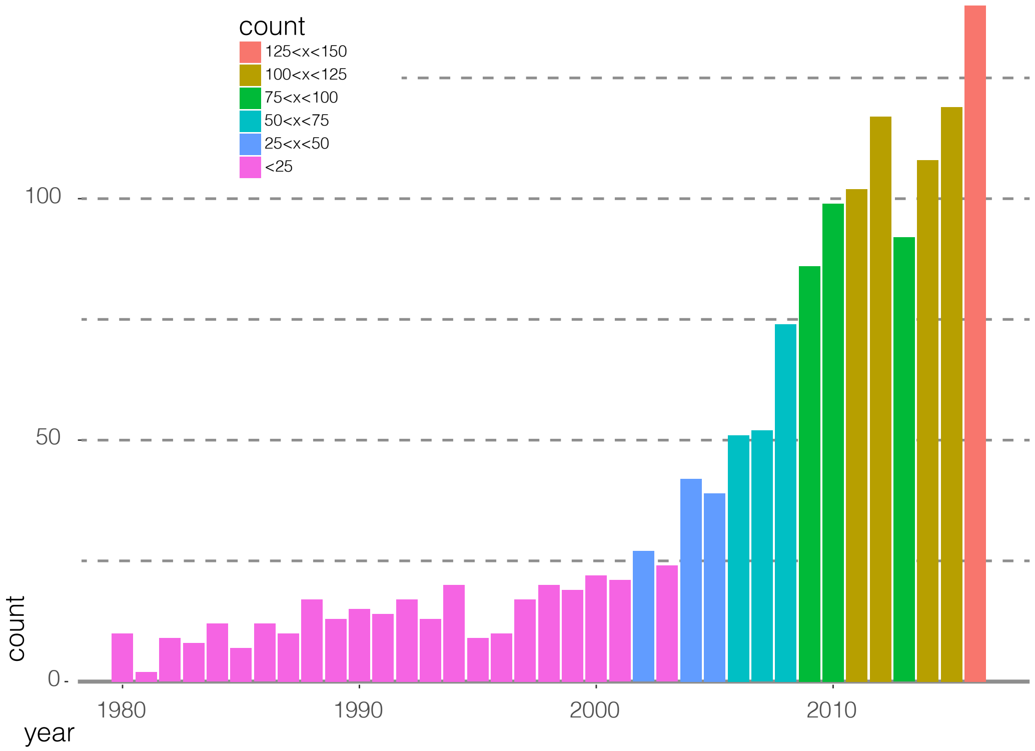

Supplemental Figure 1. Frequency of publications regarding *Cyrtodactylus* since 1980. Species descriptions, both integrative and morphology-only, have contributed considerably to the uptick in publications since 2000. Publication occurrences were pulled from Google Scholar, using a custom python script made available at https://github.com/Pold87/academic-keyword-occurrence . This excludes search results of citations only.
